# Contrast-Enhanced Mammography (CEM) Capability to Distinguish Molecular Breast Cancer Subtypes

**DOI:** 10.3390/biomedicines10102384

**Published:** 2022-09-24

**Authors:** Elzbieta Luczynska, Tomasz Piegza, Joanna Szpor, Sylwia Heinze, Tadeusz Popiela, Jaromir Kargol, Wojciech Rudnicki

**Affiliations:** 1 Department of Electroradiology, Jagiellonian University Medical College, 31-008 Cracow, Poland; 2 Department of Radiology, 5th Military Clinical Hospital in Cracow, 30-901 Cracow, Poland; 3 Department of Pathomorphology, Jagiellonian University Medical College, 30-688 Cracow, Poland; 4 Department of Radiology, Maria Sklodowska-Curie National Research Institute of Oncology in Cracow, 31-115 Cracow, Poland; 5 Department of Radiology, Jagiellonian University Medical College, 30-688 Cracow, Poland; 6 Institute of Medical Sciences, Medical College of Rzeszów University, 35-959 Rzeszów, Poland

**Keywords:** breast cancer, CEM, MRI, receptors, precision medicine

## Abstract

With breast cancer ranking first among the most common malignant neoplasms in the world, new techniques of early detection are in even more demand than before. Our awareness of tumors’ biology is expanding and may be used to treat patients more efficiently. A link between radiology and pathology was searched for in our study, as well as the answer to the question of whether a tumor type can be seen on contrast-enhanced mammography and if such knowledge may serve as part of precision medicine.

## 1. Introduction

Breast cancer is the most frequent cancer in the world, taking into account women and men together. It is said to have dethroned the former leader, lung cancer, considering the malignant tumor incidence worldwide in 2020 [1]. Since breast cancer diagnostics is a difficult issue, it requires constant development. New methods that allow the detection of early-stage breast cancers are being sought [2,3,4]. Over the years, several artificial intelligence models that analyze medical images have also been developed to aid clinicians in diagnosing breast cancer. These methods facilitate early detection and verification of the lesion, which enable accurate treatment [5,6,7]. Hence, it is not only the detection of the lesion that is important, but also its grading and biological type assessment. Currently, precision medicine is being used more often. It provides an individual approach to each patient, allowing the treatment to be “tailored” to their needs. Contrast-enhanced mammography (CEM) is one of these new methods. Despite being a new diagnostic method, research and publications show that it is comparable with breast MRI [8,9,10,11]. Currently, CEM is used as a substitute for MRI if it is not available or if there are any contradictions (such as metal in the body, a cardio-stimulator that cannot be used in MRI, allergy to gadolinium contrast agents or claustrophobia). Additionally, the availability of terms for the examinations is considered (in correlation with the patient’s menstrual cycle) when choosing the method. CEM and MRI are used in breast cancer diagnosis when we need to know whether it is a lobular cancer, when we suspect recurrent disease in the previously treated breast, when we need to verify multifocal cancer or if there is an inconsistency in the US and MG results. In breast cancer treatment, we use CEM and/or MRI to monitor the response to neoadjuvant chemotherapy. We also choose contrast-enhanced imaging methods in patients with a high risk of breast cancer (such as a dense breast type, a family history of breast cancer or genetic mutations).

Currently, breast cancers may be divided into surrogate biological subtypes on the basis of four markers assessed in histological examination. The markers include estrogen receptor (ER) expression, progesterone receptor (PR) expression, human epidermal growth factor receptor 2 (HER2) expression and the Ki67 proliferation index (Ki67). According to St. Gallen’s consensus, the subtypes are: luminal A (positive hormone receptors, with strong expression, negative HER2 and low-level Ki67, usually Grade 1 or Grade 2); luminal B-like (HER2-negative, progesterone receptor-positive, but the expression level is lower, clearly high Ki67, usually Grade 3); HER2-positive (depending on positive or negative hormone receptor expression—luminal B or non-luminal, usually Grade 3); triple-negative breast cancer (estrogen receptor-negative, progesterone receptor-negative and HER2-negative, usually Grade 3) [12].

The choice of local and systemic treatment methods on individual cancer stages is based on clinical and pathomorphological evaluation, with regard to histologic type, cancer grade, ER/PR expression, HER2 state, primary tumor stage, axillary lymph node stage, presence, location and extension in distant organs, discomfort resulting from the tumor, conditions posing an immediate risk to life, time between primary treatment and tumor recurrence, type of previous treatment and response to it, menopause state and the patient’s age, fitness state, past and coexisting diseases and their treatment and preferences.

An attempt to establish the tumor type before the biopsy procedure based on the tumor CEM image may enable faster treatment implementation, while the choice of optimal imaging method for treatment response evaluation, with regard to the biological type of the tumor, could optimize the surgical treatment choice [13,14,15,16].

## 2. Materials and Methods

This prospective study involved patients who underwent CEM between January 2020 and June 2021 and was approved by the Bioethics Committee at the District Medical Chamber in Cracow.

**Figure 1:**
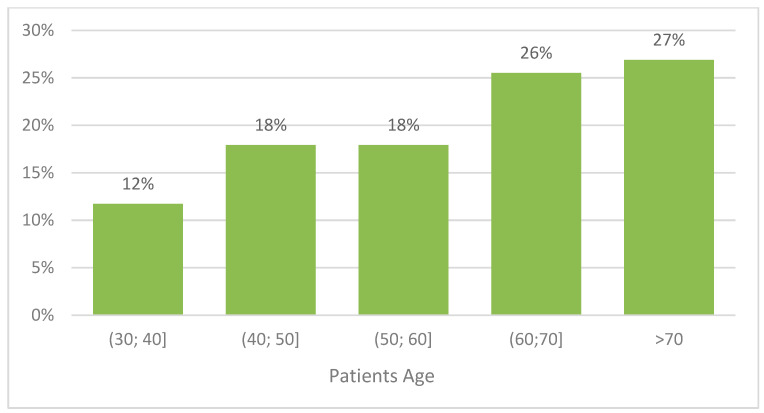
Age distribution of the analyzed patients.

Primarily, there were 283 patients included in the study group. Enhancement on CEM was not shown in 25 patients, and a further 94 patients did not receive confirmation of malignancy on histopathology, which resulted in their exclusion from the group. Another 16 patients decided to continue treatment in another clinic. The final material includes 145 patients aged 31–89 (median 63 years). The age distribution is presented in Figure 1.

CEM was performed with a GE machine (GE Healthcare, Buc, France). Mammography imaging was performed 2 min after intravenous contrast medium administration at a dose of 1.5 mL/kg at a rate of 3 mL/s. Each breast was examined in standard bilateral craniocaudal (CC) and mediolateral oblique (MLO) projections. The first projection in each breast was always CC. The examination begun with the healthy breast without any focal lesion detected on other imaging modalities.

**Figure 2:**
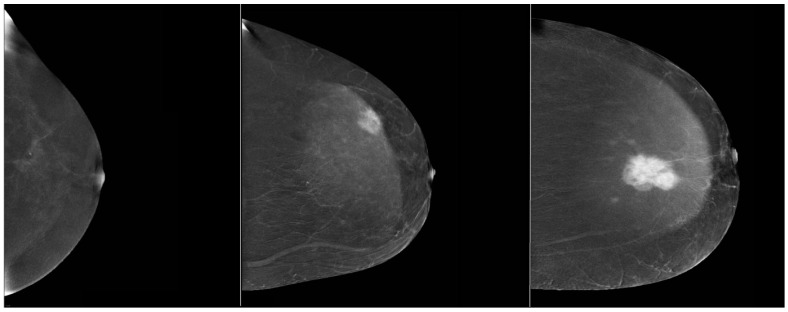
Qualitative assessment of the enhancement level on CEM (weak, medium and strong enhancement levels of suspicious breast lesions).

CEM was assessed by two radiologists, with 10 and 6 years of experience. The characteristics evaluated on CEM included the presence of pathological enhancement foci and the level of enhancement of the visible lesions with qualitative and quantitative methods. Qualitative assessment of the enhancement level on CEM was divided into strong, moderate and weak, which is presented in Figure 2.

**Figure 3:**
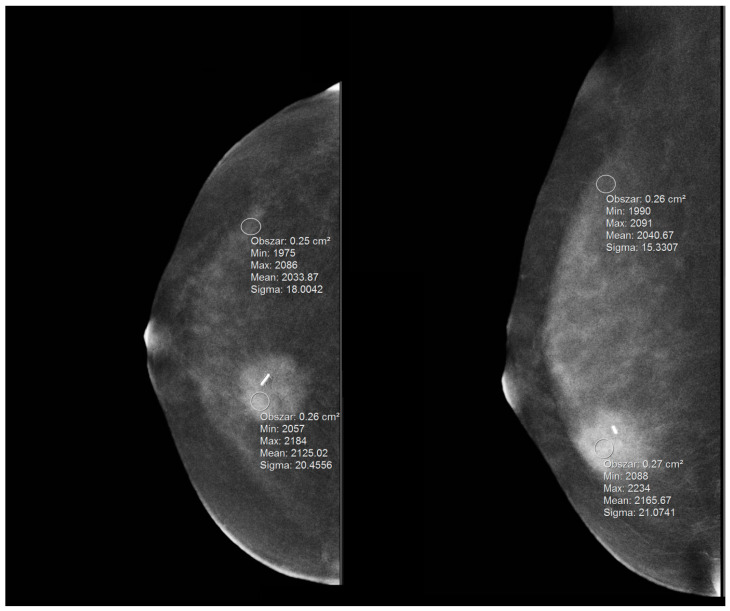
The method of measurement with the ROI within the focal lesion and BPE, presented on MLO and CC projections.

Contrast enhancement of the lesion was also subjected to a quantitative analysis. In order to enable such an analysis, a region of interest (ROI) measurement tool was used. The ROI with an elliptical shape was placed manually in the most homogeneous area of the suspicious lesion. The optimal placement and shape of the ROI depended on the homogeneity of the structure (the bigger, the better), shape and extent of contrast enhancement. The ROI area never extended beyond the contour of the evaluated enhancing lesion. The second ROI was placed beyond the pathological lesion, over the most homogeneous breast tissue area. Such a placement of the ROI was supposed to reduce the possibility of different levels of parenchyma enhancement occurrence and to be more representative for the background signal. The method of ROI placement over the individual areas is presented in Figure 3.

ROI values were assessed separately for CC and MLO projections. ROI diameters were similar within the enhancing lesion and beyond it, with this similarity maintained in both projections. Both the average signal and standard deviation were recorded while evaluating the ROI values in BPE. On the basis of the data collected, the relative enhancement parameters %RS and SDNR were measured for every lesion. %RS, which is the percentage difference between the enhancing lesion and its background, and SDNR, which is the signal-to-noise ratio, were calculated using the following formulas:

(1) $$\%\mathrm{RS}=\frac{\mathrm{ls}-\mathrm{bs}}{\mathrm{bs}}\times 100\%$$

(2) $$\mathrm{SDNR}=\frac{\mathrm{ls}-\mathrm{bs}}{\sigma \mathrm{bs}}$$

where:

ls—signal in the lesion;
bs—signal in the background (parenchyma);
σ—standard deviation of the signal in the parenchyma.

Subsequently, the patients underwent ultrasound examination (US). All patients with suspicious lesions on US had a biopsy under US guidance, whereas the patients with lesions not visible on US but visible on MG had the biopsy performed under MG guidance or DBT (digital breast tomosynthesis). In 4 patients, the biopsy was performed under MRI guidance because their lesions were confirmed on MRI but not on MG or US.

### 2.1. Histopathological Examination

Histopathological examinations were performed in the pathology department of the University Hospital for all patients. The examination was conducted after core biopsy or VABB, with each specimen undergoing formalin fixation followed by paraffin embedding. Tumor parameters were assessed by microscopically examining the sections stained with hematoxylin and eosin. The histological tumor type, cancer grade, ER, PR and HER2 expression and Ki67 level were determined in all patients.

Immunohistochemistry (IHC) for estrogen receptor (ER), progesterone receptor (PR) and Ki67 protein was performed according to the protocol routinely used in the laboratory. The selected blocks were cut into 4 μm-thick sections. For ER (clone 6F11, 1:100, Novocastra, Leica Biosystems, Nußloch, Germany, incubation time 30 min), PR (clone PgR636, 1:100, Dako, Carpinteria, CA, USA, incubation time 60 min) and Ki67 (clone MIB-1, 1:100, Dako, USA, incubation time 30 min), antigen retrieval was performed by incubating the slides in citrate buffer (pH 6.0; 0.01 M) at 97 °C in a water bath for 40 min. An UltraVision Quanto detection system (Lab Vision, ThermoScientific, Waltham, MA, USA) and 3,3′-diaminobenzidine as the chromogen were used, and the slides were counterstained with Mayer hematoxylin (Thermo Fisher Scientific, USA) and coverslipped. Immunohistochemistry for HER2 (PATHWAY 4B5, Ventana Medical Systems Inc., Tucson, AZ, USA) was performed on a BenchMark BMK Classic autostainer (Ventana, USA) using an UltraVIEW DAB Detection Kit (Ventana Medical Systems Inc., USA). For specimens with HER2 status 2+ in immunohistochemistry, fluorescence in situ hybridization (FISH) was conducted. FISH was performed using a ZytoLight SPEC ERB2/CEN17 Dual Color Probe Kit (ZytoVision, Bremerhaven, Germany) according to the manufacturer’s protocol. The green Locus-Specific Identifier (LSI) HER-2/neu and orange Centromere Enumeration Probe (CEP 17) signals were counted on a fluorescence microscope equipped with specific filter sets. HER2 status was assessed according to the 2018 ASCO/CAP guidelines. When the ratio of HER2/CEP17 was ≥2.0 with an average HER2 signals/cell of ≥4.0, the HER2 status was classified as positive. When the ratio of HER2/CEP17 was < 2.0 with an average HER2 signals/cell of ≥6.0, FISH was recounted with an additional observer. If the ratio remained <2.0 with ≥6.0 HER2 signals/cell, the diagnosis was HER2-positive. When the ratio of HER2/CEP17 was <2.0 with an average HER2 signals/cell of < 4.0, the HER2 status was considered negative. When the ratio of HER2/CEP17 was <2.0 with an average HER2 signals/cell of ≥4.0 and <6.0, FISH was recounted with an additional observer. If the ratio remained ≥4.0 and <6.0 HER2 signals/cell, the diagnosis was HER2-negative with an appropriate comment.

When the ratio of HER2/CEP17 was ≥2.0 with an average HER2 signals/cell of <4.0, FISH was recounted with an additional observer. If the ratio remained ≥4.0 and <6.0 HER2 signals/cell, the diagnosis was HER2-negative with an appropriate comment.

### 2.2. Statistical Methods

Continuous variable statistics (age, enhancement parameters) are shown as medians with quartiles (25% and 75%) as they did not present a normal distribution. Thus, to compare variables within the groups, a non-parametric Mann–Whitney U test was applied. The frequency in percentage is presented for qualitative variables. To assess the relations between qualitative variables, contingency tables were used. Cramer’s V contingency coefficient (ϕ) and the chi-square independence test were counted. The correlation between the variables was considered weak if 0.10 ≤ ϕ < 0.20, moderate if 0.20 ≤ ϕ < 0.40, relatively strong if 0.40 ≤ ϕ < 0.60 and strong if 0.60 ≤ ϕ < 0.80. The significance level of *p* < 0.05 was adopted for all tests.

## 3. Results

Overall, 145 lesions diagnosed in 142 patients (including double lesions in 3 patients) were subjected to analysis. Within the group, carcinoma was found in 130 (90%) patients, including 109 ductal invasive carcinomas (75% of patients), and lobular invasive carcinoma in 21 (14%) patients. Intraductal carcinoma was present in 15 (10%) patients. Basic statistics of the lesions are presented in Table 1.

Within the examined group, more luminal A cancers were determined, at 50%, while the least numerous group was represented by HER2+ non-luminal patients, at only 6%. Due to the multiplicity of the biological subtype groups, the cases were divided into two groups: luminal (luminal A and luminal B) and non-luminal (HER+, TNBC (triple-negative breast cancer)). Material analysis revealed the correlation between patients’ age and Ki67 value, as shown in Table 2.

Parenchyma enhancement was higher for cancers not showing ER/PR expression and Ki67-low (*p* < 0.02). There was no difference in parenchyma enhancement between HER2-positive and -negative tumors. The absolute enhancement value was similar in hormone-negative and -positive cases as well as in Ki67-low and Ki67-high tumors, while it was significantly higher for HER2-negative tumors. Relative enhancement measurements were significantly statistically lower in the case of HER2-negative tumors (*p* < 0.03). The above correlations are presented in Table 2 and Table 3.

Subsequently, the CEM results were compared with the biological subtypes of breast cancers. The distribution of individual parameters of the lesions related to biological lesion types (luminal, non-luminal) is shown in Table 2. Mean parenchyma enhancement was higher in the case of non-luminal lesions (*p* = 0.012), while relative enhancement coefficients were lower for non-luminal lesions. This relation was statistically significant only for the values of SDNR and %RS in the CC view (*p* < 0.013). However, the absolute enhancement value (mean enhancement) was almost identical in the case of luminal and non-luminal lesions (median: 2090.5 and 2091, respectively). This is shown in Table 4.

Based on the parenchyma enhancement values and relative enhancement measures (SDNR and RS in the CC view), it is possible to determine whether the lesions are luminal or non-luminal (*p* < 0.0131). The optimal cut-off points are, respectively, 3.26 and 2.49, with parenchyma enhancement being a determinant (the chances of luminal lesion incidence diminish with the increase in the parenchyma enhancement value). The sensitivity and specificity parameters shown below allow for differentiating between luminal and non-luminal lesions for the counted cut-off points. The highest sensitivity value in differentiating between luminal and non-luminal lesions was attributed to parenchyma enhancement, with 0.741, while the specificity value at the same level of SNDR and RC in CC was 0.519.

## 4. Discussion

In order to predict the prognosis of the disease and plan the most effective treatment, it is crucial to assess and categorize tumors into adequate immunohistochemical subtypes, basing on molecular studies including the Ki67 proliferation index and many other biomarkers and tools.

Among many imaging methods available in breast cancer diagnostics, CEM is one of the latest. Contrast administration applied in this method facilitates neoangiogenesis assessment within the tumor. The problem is, however, that both the tumor and breast parenchyma show enhancement after contrast administration. Nevertheless, a number of publications confirm that CEM is a method comparable with MRI in terms of sensitivity and specificity as well as parenchyma enhancement.

The relative coefficients of enhancement were lower for non-luminal lesions, and this relation was only statistically significant (*p* < 0.013) in the CC projection (for SDNR and %RS values). The results are surprising since the CC projection was the first projection performed in our patients after contrast medium administration and enabled us to obtain statistical significance, confirming the statement that the relative enhancement coefficients were lower for non-luminal lesions. This may be related to the time interval between contrast administration and image acquisition. It is known that neoplastic lesions enhance faster on MRI than benign lesions and parenchyma, with the highest intensity at 2 min after contrast administration, followed by the wash-out effect—fast enhancement decline. This may be the reason why, in the MLO projection performed 4–5 min after contrast administration, neoplastic lesion enhancement is so weak that the relation disappears.

The absolute enhancement value was similar for ER- and PR-negative and -positive tumors as well as Ki67-low and Ki67-high tumors, while for HER2, it was significantly higher for HER2-negative tumors. In the case of this receptor (positive value), all relative enhancement measurements were statistically significantly lower for HER2 (*p* < 0.03). In the case of ER and PR, the relative enhancement for the CC projection was higher for positive receptor values (*p* < 0.026). One study revealed that the enhancement intensity in CEM images for ER- or PR-positive lesions was weaker than in CEM images of negative lesions, while HER2-positive lesions showed stronger enhancement than HER2-negative lesions. This may be explained by the higher aggressiveness of HER2-enriched tumors.

Patients with Ki67-low tumors were older (*p* = 0.00) than patients with Ki67-high tumors. This relation is confirmed in studies by de Gregorio et al. [17] and Erić et al. [18]. However, in Boughey et al. [19], a relation between patients’ age and Ki67 was not found. Thus, this issue requires further analysis on a more numerous population of patients.

The results for BPE (breast parenchyma enhancement) should also be discussed. It is assumed that this phenomenon may affect the disease course and treatment results, as determined by Bauer et al. [20]. In one of the studies concerning the analysis of parenchyma enhancement on MRI, the authors observed that the average BPE pattern was only significantly more common in luminal B (HER2-). Moderate and marked BPE prevailed over minimal and mild types in triple-negative cancers. Among all patients with mild BPE, luminal B tumors (HER2-) were significantly higher (*p* = 0.0001). Among all patients with marked BPE, triple-negative subtypes were significantly higher (*p* = 0.0074).

Our study showed that the average parenchyma enhancement was higher in non-luminal lesions (*p* = 0.012). Parenchyma enhancement was higher for negative values of ER, PR and Ki-67 (*p* < 0.02).

Taking into consideration the variable tumor appearance depending on their biological type, we assume that it is possible to choose an optimal imaging modality for treatment response monitoring of a given patient, in accordance with precision medicine principals, in order to boost the chance of the patient’s recovery. Currently, MRI has proven its effectiveness in monitoring response to treatment [21]. Nevertheless, CEM is also assumed to be highly effective in this field [22]. Further evaluation is necessary to check whether MRI and CEM are more adequate for certain cancer types.

It is worth mentioning that in the growing population of transgender patients, hormone-dependent tumors represent half of the breast neoplasms, so the possibility to predict and monitor their optimal treatment is becoming more and more desirable [23].

This study has some limitations. The most important one is the limited number of patients, which prevented us from establishing the statistical significances in separate subgroups. The second limitation is the fact that the only characteristic taken into account while assessing the enhancing lesions on CEM was the contrast enhancement level, whereas the comparison of other characteristics such as the size, margin or homogeneity of the lesion was ignored. In our further work, we will consider the possibility of using radiomics (a new concept that has been functioning in medicine for only a few years; this idea relies on processing innumerable quantities of metadata acquired from every examination, followed by extraction thereof from relevant imaging examinations, by means of appropriate created algorithms) and convolutional neural networks (CNNs—a type of artificial neural network used in image recognition and processing that is specifically designed to process pixel data) for extracting discriminant information from medical images. As a matter of fact, both of them have been extensively employed to analyze breast images of different natures, including ultrasound, CESM and MR images, achieving high performances [24,25,26,27,28,29,30,31].

Another limitation is the lack of correlation between the mammographic characteristics of the lesions, e.g., microcalcification presence with the enhancement level and biological factors. The last one pertains to the fact that the analysis did not include factors such as metastasis to the distant organs, survival rate or local recurrence. These factors may reflect the level of a tumor’s malignancy.

## 5. Conclusions

CEM shows potential in distinguishing between some subtypes of breast cancer, and therefore it can be a useful tool for monitoring treatment and surgery planning. In order to improve our knowledge, further studies on this topic are definitely needed.

## Figures and Tables

**Table 1:** Clinicopathological characteristics of the lesions and patients (abbreviations: estrogen receptor (ER) expression, progesterone receptor (PR) expression, human epidermal growth factor receptor 2 (HER2) expression, Ki67 proliferation index (Ki67), ductal carcinoma in situ (DCIS), carcinoma of no special type (Ca NST), triple-negative breast cancer (TNBC)).

Characteristic	Description	Proportion (%)
Age	<45 years	34/145 23%
	≥45 years	111/145 77%
Histological type	DCIS	15/145 10%
	Ca lobular	21/145 14%
	Ca NST	109/145 75%
Malignancy	No	15/145 10%
	Yes	130/145 90%
Molecular subtypes	Luminal	118/145 81%
	-Luminal A	73/145 50%
	-Luminal B	45/145 31%
	HER2, TNBC	27/145 18%
	-HER2	9/145 6%
	-TNBC	18/145 12%
ER	Negative	28/145 19%
	Positive	117/145 81%
PR	Negative	33/145 23%
	Positive	112/145 77%
HER2	Negative	98/130 75%
	Positive	32/130 25%
Ki67	Low	63/109 58%
	High (>20%)	46/109 42%
Enhancement degree	Weak	19/145 13%
	Medium	46/145 32%
	Strong	80/145 55%

**Table 2:** Relation between CEM and ER and PR receptor status. Significant *p* values are in bold.

Continuous Variables	ER+ (N = 117)	ER- (*N* = 28)	*p* Value	PR+ (*N* = 112)	PR- (*N* = 33)	*p* Value
Age	63 (48, 72)	57.5 (42, 69)	0.144	63 (48, 71.8)	59 (42, 70)	0.230
Mean Enhancement	2089.5 (2055.8, 2129.5)	2091.3 (2071.8, 2115.8)	0.397	2092.3 (2056, 2130.8)	2090.5 (2060.5, 2111.5)	0.354
Mean BPE	2020.9 (2014, 2035.6)	2029.3 (2019.5, 2039.6)	**0.014**	2020.7 (2014, 2034.2)	2032.2 (2020.5, 2039.2)	**0.004**
SDNR_CC	4.4 (1.8, 6.9)	3.1 (1.2, 4.8)	**0.016**	4.6 (1.8, 6.9)	2.9 (1.2, 4.5)	**0.005**
SDNR_MLO	4.3 (1.9, 6.8)	5.2 (2.5, 6.9)	0.221	4.5 (1.8, 7.1)	4.1 (2.5, 6.5)	0.368
SDNR	4.5 (2.2, 6.9)	3.8 (2.3, 5.4)	0.228	4.5 (2.2, 7)	3.1 (2.2, 5.3)	0.095
%RS_CC	3.2 (1.3, 5.2)	2.4 (0.8, 3.7)	**0.026**	3.4 (1.3, 5.2)	2.1 (0.8, 3.4)	**0.005**
%RS_MLO	3.3 (1.5, 5.1)	3.9 (1.9, 4.9)	0.179	3.4 (1.4, 5.3)	3.4 (1.7, 4.8)	0.390
%RS	3.4 (1.6, 5)	2.8 (1.8, 3.8)	0.265	3.5 (1.7, 5)	2.6 (1.6, 3.8)	0.085

**Table 3:** Relation between CEM and HER and Ki67 status.

Quantitative Variable	HER+ (*N* = 32)	HER- (*N* = 98)	ϕ	*p* Value	Ki-67 (High) (*N* = 46)	Ki-67 (Low) (*N* = 63)	ϕ	*p* Value
Age			0.07	0.59			0.28	0.006
<45 ≥45	9/32 28% 23/32 72%	21/98 21% 77/98 79%			18/46 39% 28/46 61%	9/63 14% 54/63 86%		

**Table 4:** Distribution of continuous radiological findings according to molecular subtypes.

Continuous Variables	Luminal Subtype (*N* = 118)	Non-Luminal Subtype (*N* = 27)	*p* Value
Age	63 (48, 72)	56 (40, 69)	0.107
Mean Enhancement	2090.5 (2056, 2130.3)	2091 (2057.5, 2112)	0.47
Mean BPE	2020.9 (2014, 2035.5)	2032 (2018.5, 2039.6)	**0.012**
SDNR_CC	4.5 (1.8, 6.8)	2.9 (1, 4.8)	**0.011**
SDNR_MLO	4.5 (1.9, 6.9)	4.1 (2.4, 6.4)	0.457
SDNR	4.5 (2.2, 6.9)	3.1 (2.2, 5.4)	0.107
%RS_CC	3.3 (1.3, 5.2)	2.3 (0.7, 3.6)	**0.013**
%RS_MLO	3.4 (1.5, 5.3)	3.4 (1.8, 4.9)	0.37
%RS	3.4 (1.7, 5)	2.7 (1.7, 3.8)	0.13

Data are presented as the median values with quartile intervals (25%, 75%). Significant *p* values are in bold.
